# Predictive assessment in pharmacogenetics of *XRCC1* gene on clinical outcomes of advanced lung cancer patients treated with platinum-based chemotherapy

**DOI:** 10.1038/srep16482

**Published:** 2015-11-20

**Authors:** Zhengrong Yuan, Jiao Li, Ruiqi Hu, Yang Jiao, Yingying Han, Qiang Weng

**Affiliations:** 1 College of Biological Science and Technology, Beijing Forestry University, Beijing 100083, People’s Republic of China; 2 Institute of Medicinal Plant Development, Chinese Academy of Medical Sciences & Peking Union Medical College, Beijing 100193, People’s Republic of China; 3 National Animal Husbandry Service, Beijing 100125, People’s Republic of China

## Abstract

Published data have shown inconsistent results about the pharmacogenetics of *XRCC1* gene on clinical outcomes of advanced lung cancer patients treated with platinum-based chemotherapy. This meta-analysis aimed to summarize published findings and provide more reliable association. A total of 53 eligible studies including 7433 patients were included. Patients bearing the favorable TrpTrp and TrpArg genotypes of Arg194Trp were more likely to better response rates to platinum-based chemotherapy compared to those with the unfavorable ArgArg genotype (TrpTrp+TrpArg vs. ArgArg: odds ratio (OR) = 2.02, 95% CI, 1.66–2.45). The GlnGln and GlnArg genotypes of Arg399Gln were significantly associated with the poorer response rates compared to those with the ArgArg genotype (GlnGln +GlnArg vs. ArgArg: OR = 0.68, 95% CI, 0.54–0.86). The GlnGln genotype might be more closely associated with shorter survival time and higher risks of death for patients (GlnGln vs. ArgArg: hazard ratio (HR) = 1.14, 95% CI, 0.75–1.75). Our cumulative meta-analyses indicated a distinct apparent trend toward a better response rate for Arg194Trp, but a poorer response rate in Arg399Gln. These findings indicate a predictive role of *XRCC1* polymorphisms in clinical outcomes. The use of *XRCC1* polymorphisms as predictive factor of clinical outcomes in personalized chemotherapy treatment requires further verification from large well-designed pharmacogenetics studies.

Lung cancer, a major serious public health problem, is one of the most common malignant tumors and has become the leading cause of cancer-related deaths in the world^1^, with more than one million deaths from this disease annually^2^,^3^. There are two main types of lung cancer: non-small cell lung cancer (NSCLC) and small cell lung cancer (SCLC), of which NSCLC accounts for approximately 80%–85% and nearly 70% of patients present with the locally advanced stages (such as stage IIIB or IV) or metastatic disease at the time of diagnosis, losing the opportunity of surgical resection and making curative surgery impossible^3^,^4^,^5^,^6^,^7^,^8^,^9^. Although intensive effort has been made to improve the efficacy of lung cancer diagnosis and therapy in the last decades, the overall five-year survival rate still remains only about 15% and even lower in China^1^,^2^.

The treatment of lung cancer patients is surgery for early stages, whereas chemotherapy regimen is the main conventional therapeutic method for locally advanced stages and metastatic cancers. Currently, the platinum-based chemotherapy is one of the most extensively accepted and used treatments in advanced lung cancer patients, which has been shown to improve overall survival (OS)^10^,^11^,^12^. However, individual in clinical practice, the therapeutic efficacy of chemotherapy varies remarkably among different individuals, with a response rate from 26% to 60% in lung cancer patients^13^,^14^. Some individuals response to the chemotherapy, while others confer intrinsic or acquired drug resistance^15^. It has been well recognized that genetic factors are considered to play an important role in disease development, drug response, treatment effectiveness, and survival of lung cancer, thus affect the prognosis of patients^8^,^14^. Seeking optimal therapeutic and prognostic biomarkers, which could improve prognostic and predictive assessment accuracy to help early detection, better chemotherapeutic agents, drug sensitivity, developing personalized cancer treatment and patient-tailored chemotherapy, and eventually achieving better outcomes for patients^7^,^8^,^16^. The therapeutic efficacy of platinum-based chemotherapy treatment remains a research hotpot in this field^7^,^8^,^16^. However, the reliable biomarkers are still lacking in the clinical practice.

It has been speculated that the single nucleotide polymorphisms (SNPs) or mutations occurring in the DNA repair pathways may alter gene expression and the activity of DNA repair^17^,^18^, thus influence the effectiveness of cancer therapy, prognosis and survival of patients^19^. The DNA repair pathways mainly include the base excision repair (BER), nucleotide excision repair (NER), DNA double strand break repair (DDSBR), and mismatch repair (MMR)^20^. The X-ray repair cross-complementing group 1 (*XRCC1*) gene, located on chromosome 19q13.2, has long been recognized as a central role in BER pathway by interacting with other DNA repair proteins, acting as a “scaffold” in both DNA single-strand break repair and BER activities^21^,^22^,^23^,^24^,^25^. It has been proposed that the XRCC1 protein is critical for repairing other types of DNA damage induced by the platinum-based anticancer drugs (such as cisplatin (DDP) and carboplatin (CBP))^26^, also including DNA double-strand breaks^27^,^28^. Therefore, genetic variants in *XRCC1* gene might modulate DNA repair capacity, and hence markedly influence the anticancer effect of platinum drugs, the efficacy of platinum-based chemotherapy treatment and the prognosis and survival of patients. Although a great number of studies have extensively investigated the pharmacogenetics of *XRCC1* genetic variants (such as rs1799782, C → T, arginine (Arg)194 tryptophan (Trp), exon 6; rs25489, G → A, Arg280 histidine (His), exon 6; rs25487, G → A, Arg399glutanine (Gln), exon 10; rs3213245, C→T, T-77C, 5′-untranslated region (5′-UTR); and rs3213239 (insertion variation GGCC), http://www.ncbi.nlm.nih.gov/projects/SNP) on clinical outcomes of platinum-based chemotherapy in advanced lung cancer patients, the observed results from these studies remain conflicting rather than conclusive^6^,^7^,^10^,^29^,^30^,^31^,^32^,^33^,^34^,^35^,^36^,^37^,^38^,^39^,^40^,^41^,^42^,^43^,^44^,^45^,^46^,^47^,^48^,^49^,^50^,^51^,^52^,^53^,^54^,^55^,^56^,^57^,^58^,^59^,^60^,^61^,^62^,^63^,^64^,^65^,^66^,^67^,^68^,^69^,^70^,^71^,^72^,^73^,^74^,^75^,^76^,^77^,^78^. Because a single study may be too underpowered to detect slight effects of the genetic variants on cancer, especially when the sample size is relatively small, the meta-analysis could provide more comprehensive and reliable conclusions through systematically summarizing existed data. There are several meta-analyses reported the inconsistent results for evaluating the associations between the *XRCC1* gene polymorphisms and response to platinum-based chemotherapy in lung cancer^14^,^15^,^16^,^79^,^80^,^81^. These meta-analyses may have not enrolled all of available studies which are published before or after their meta-analysis, thus may have biased the conclusions. Therefore, in the present study, we conducted an update system review and meta-analysis to combine all available publications on pharmacogenetic studies, and derived more precise and comprehensive assessment on the reliable associations of the commonest *XRCC1* genetic polymorphisms on the efficacy and clinical outcomes of advanced lung cancer patients treated with platinum-based chemotherapy.

## Results

### General characteristics of eligible studies

**Figure 1:**
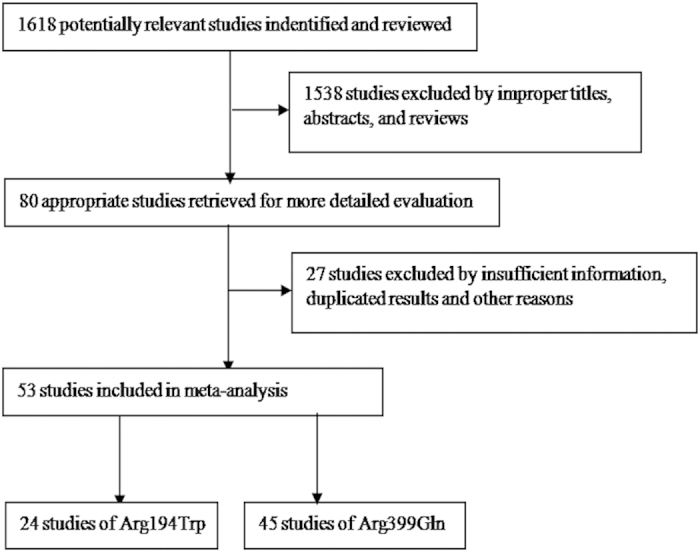
The flow chart of literatures search and selection of included studies.

Overall, 1618 publications were retrieved by systematic literature search using different combinations of key terms. According to our inclusion and exclusion criteria, 53 eligible studies were included for evaluating the data pool for our systematic review (Fig. 1), altogether 7433 advanced lung cancer patients. The general characteristics of the included publications are shown in Table 1. Only two *XRCC1* genetic polymorphisms, rs1799782 (Arg194Trp) and rs25487 (Arg399Gln) were finally enrolled in this meta-analyses. Other *XRCC1* genetic polymorphisms were not included because of insufficient numbers of relevant publications on each polymorphism, such as three studies with rs25489 (Arg280His), two studies with rs3213245 (T-77C), one study with rs3213239 (insertion variation GGCC). 24 of the included studies were reported in the Arg194Trp genetic polymorphism, and 45 were reported in the Arg399Gln genetic polymorphism. Seven of the included studies were conducted on Caucasian patients, and 46 were conducted on Asian patients. 22 studies were published in English, and 31 studies were published in Chinese. There were 46 studies reported the objective response rate (ORR), 22 studies reported the OS and hazard ratios (HRs), 20 studies reported the median survival time (MST), four studies reported the median time to progression (TTP), and 11 studies reported the median progression-free survival (PFS) (Tables 1 and 2). The sample size ranged from 45 to 460 advanced lung cancer patients. The quality score (QS) of the included studies is summarized in Tables 1, and ranged from 6 to 23. Among these studies, 40 studies were high quality, 13 were low quality.

### Objective response rate of *XRCC1* genetic polymorphisms

**Figure 2:**
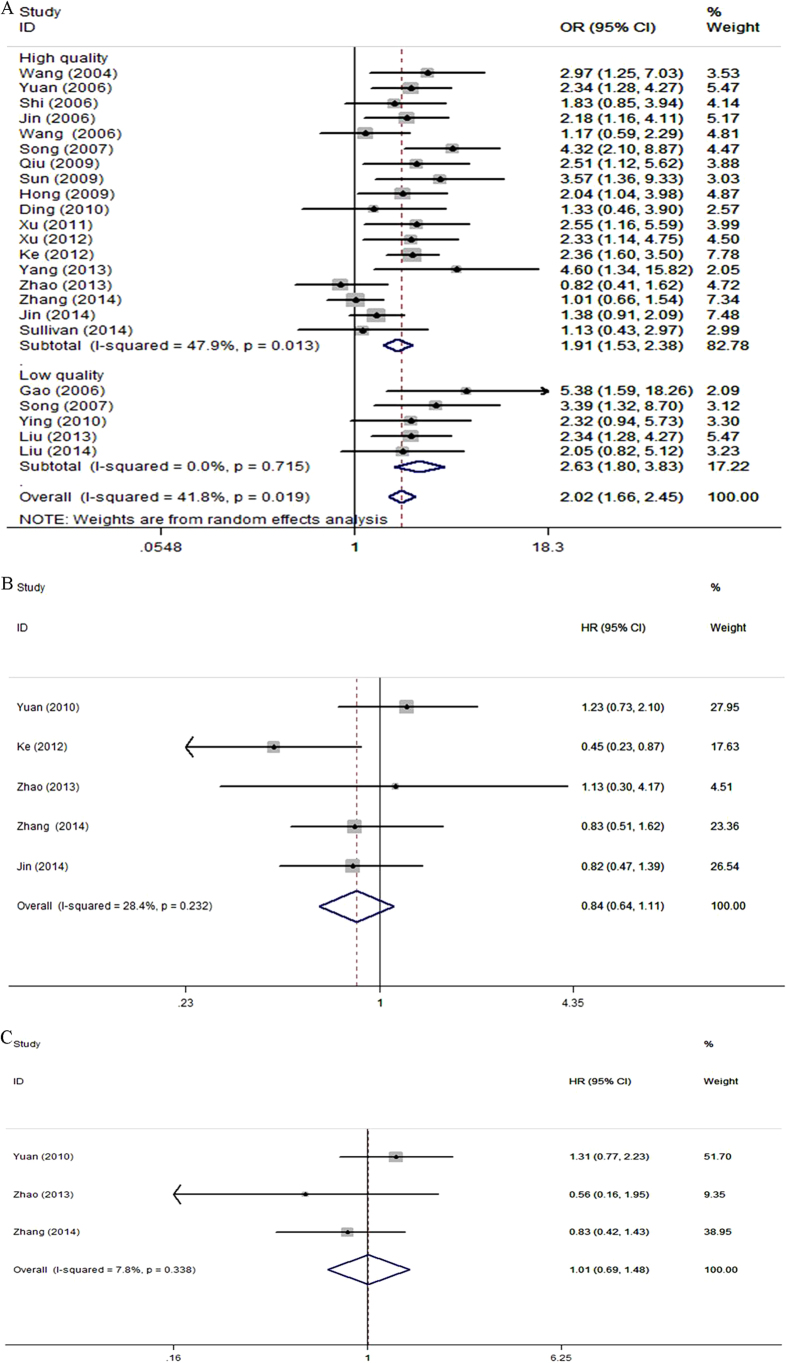
Forest plots of clinical outcomes in advanced lung cancer patients treated with platinum-based chemotherapy by the *XRCC1* Arg194Trp polymorphism. (**A**) Odds ratios (ORs) (and its 95% confidence interval (CI)) of objective response rate (ORR) stratified by study quality levels for TrpTrp+TrpArg vs. ArgArg. (**B**). Hazard ratios (HRs) (and its 95% CI) of overall survival (OS) for TrpTrp vs. ArgArg. (**C**). HRs (and its 95% CI) of median progression-free survival (PFS) for TrpTrp vs. ArgArg.

23 eligible studies including 3662 advanced lung cancer patients were qualified for the final analysis for the *XRCC1* Arg194Trp polymorphism. The results from the meta-analysis suggested that there were statistically significant associations between the *XRCC1* Arg194Trp polymorphism and ORR under all the genetic models (TrpTrp versus (vs.) ArgArg: odds ratio (OR) = 2.07, 95% confidence intervals (CIs), 1.67–2.58, P < 0.001; TrpArg vs. ArgArg: OR = 2.11, 95% CI, 1.68–2.65, P < 0.001; TrpTrp+TrpArg vs. ArgArg: OR = 2.02, 95% CI, 1.66–2.45, P < 0.001, Fig. 2A; TrpTrp vs. TrpArg+ArgArg: OR = 1.56, 95% CI, 1.27–1.91, P < 0.001; Trp vs. Arg: OR = 1.69, 95% CI,1.46–1.95, P < 0.001, Table 3). Subgroup analyses by QS suggested that, for the high quality group, the Arg194Trp polymorphism was significantly associated with ORR of advanced lung cancer patients under all the genetic models (TrpTrp vs. ArgArg: OR = 2.08, 95% CIs, 1.66–2.63, P < 0.001; TrpArg vs. ArgArg: OR = 1.96, 95% CI, 1.51–2.54, P < 0.001; TrpTrp+TrpArg vs. ArgArg: OR = 1.91, 95% CI, 1.53–2.38, P < 0.001; TrpTrp vs. TrpArg+ArgArg: OR = 1.62, 95% CI, 1.30–2.02, P < 0.001; Trp vs. Arg: OR = 1.67, 95% CI,1.39–1.99, P < 0.001, Table 3). For the low quality studies group, the significant associations between Arg194Trp polymorphism and ORR of advanced lung cancer patients were found in the heterozygote genetic model (TrpArg vs. ArgArg: OR = 2.83, 95% CI, 1.90–4.22, P < 0.001), in the dominant genetic model (TrpTrp+TrpArg vs. ArgArg: OR = 2.63, 95% CI, 1.80–3.84, P < 0.001), and in the allele genetic model (Trp vs. Arg: OR = 1.78, 95% CI,1.36–2.33, P < 0.001, Table 3). The lung cancer patients bearing the favorable 194Trp allele, TrpTrp, and TrpArg genotypes were more likely to better response rates to platinum-based chemotherapy compared to those with the unfavorable 194Arg allele, and ArgArg genotype (Table 3).

**Figure 3:**
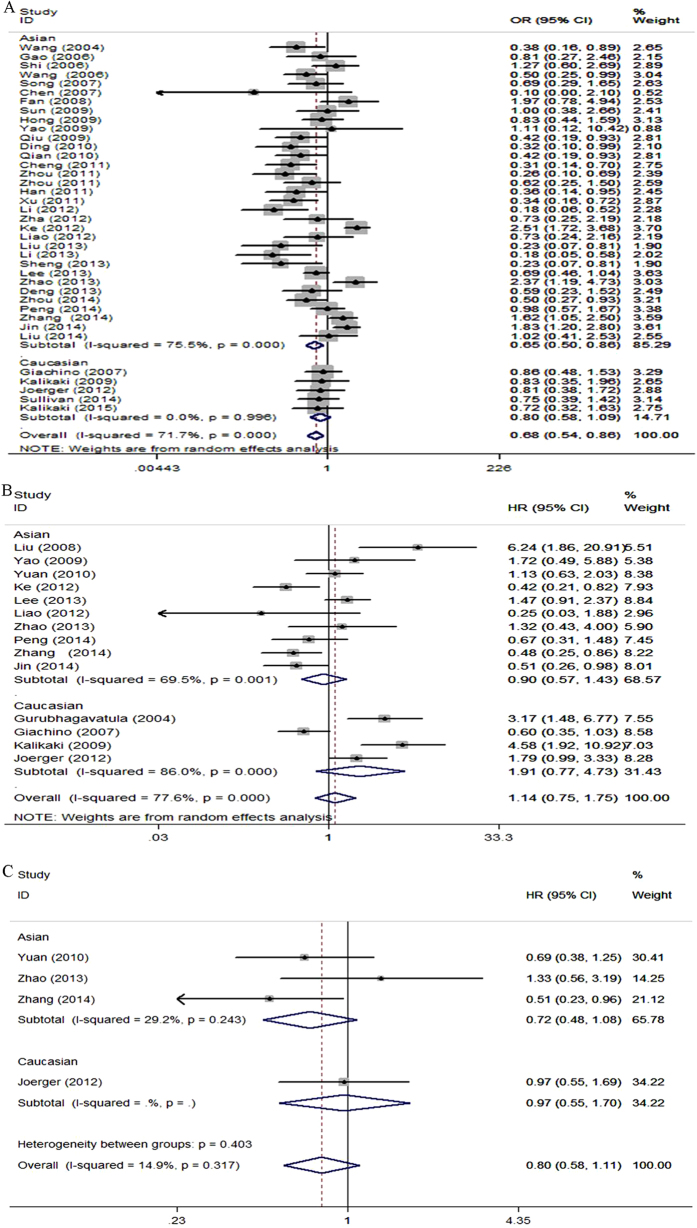
Forest plots of clinical outcomes in advanced lung cancer patients treated with platinum-based chemotherapy by the *XRCC1* Arg399Gln polymorphism. (**A**) Odds ratios (ORs) (and its 95% confidence interval (CI)) of objective response rate (ORR) stratified by ethnicity for GlnGln+GlnArg vs. ArgArg. (**B**) Hazard ratios (HRs) (and its 95% CI) of overall survival (OS) stratified by ethnicity for GlnGln vs. ArgArg. (**C**) HRs (and its 95% CI) of median progression-free survival (PFS) stratified by ethnicity for GlnGln vs. ArgArg.

There were 38 eligible studies with a total number of 5360 advanced lung cancer patients, qualified for the final analysis for the *XRCC1* Arg399Gln polymorphism. In the dominant genetic model, the GlnGln and GlnArg genotypes of *XRCC1* Arg399Gln polymorphism were significantly associated with the unfavorable ORR in advanced lung cancer patients treated with platinum-based chemotherapy compared to those with the favorable 399ArgArg genotype (GlnGln+GlnArg vs. ArgArg: OR = 0.68, 95% CI, 0.54–0.86, P = 0.001, Fig. 3A, Table 4). Subgroup analyses by stratified patient populations indicated the Arg399Gln polymorphism was not significantly associated with ORR of advanced lung cancer patients in Caucasians under all genetic models (Fig. 3A, Table 4). For the Asian group, the Arg399Gln polymorphism was also not significantly associated with ORR in four genetic models (P > 0.05, Table 4), except for dominant genetic model (GlnGln+GlnArg vs. ArgArg: OR = 0.65, 95% CI, 0.50–0.86, P = 0.002, Fig. 3A, Table 4). We also performed subgroup analysis by QS. For the high quality group, significant association between the Arg399Gln polymorphism and ORR of advanced lung cancer patients was only found in the dominant genetic model (GlnGln+GlnArg vs. ArgArg: OR = 0.72, 95% CI, 0.56–0.94, P = 0.017, Table 4). For the low quality studies group, the Arg399Gln polymorphism was statistically significantly associated with ORR of advanced lung cancer patients in the dominant genetic model (GlnGln+GlnArg vs. ArgArg: OR = 0.53, 95% CI, 0.32–0.89, P = 0.017, Table 4), in the homozygote genetic model (GlnGln vs. ArgArg: OR = 0.36, 95% CI, 0.14–0.94, P = 0.037, and in the allele genetic model (Gln vs. Arg: OR = 0.62, 95% CI, 0.43–0.90, P = 0.012, Table 4). Results suggested that the 399Gln allele may be indicative of poorer response rate to platinum-based treatment than the 399Arg allele (Table 4).

### Overall survival of *XRCC1* genetic polymorphisms

Five studies including 1559 advanced lung cancer patients were eligible for the final analysis of the relationship between the *XRCC1* Arg194Trp polymorphism and OS. The results from the meta-analysis indicated that no statistically significant relationships between the *XRCC1* Arg194Trp polymorphism and OS (TrpTrp vs. ArgArg: HR = 0.84, 95% CI, 0.64–1.11, P = 0.223, Fig. 2B; TrpArg vs. ArgArg: HR = 0.96, 95% CI, 0.79–1.16, P = 0.653; TrpTrp+TrpArg vs. ArgArg: HR = 0.93, 95% CI, 0.72–1.21, P = 0.590, Table 3). No evidence of heterogeneity with respect to predictive value was observed (Table 3).

A total of 19 studies including 3707 advanced lung cancer patients were included in the final analysis of the relationship between the *XRCC1* Arg399Gln polymorphism and OS. Variant genotypes of *XRCC1* 399 genetic polymorphism were more likely to associate with lower rates of OS and higher risks of death for advanced lung cancer patients (GlnGln vs. ArgArg: HR = 1.14, 95% CI, 0.75–1.75, P = 0.533, Fig. 3B; GlnGln vs. GlnArg: HR = 1.42, 95% CI, 1.01–2.00, P = 0.043, Table 4). The results suggested that the 399Gln allele and/or GlnGln genotype might be more closely associated with shorter survival time and higher risks of death for advanced lung cancer patients than the 399Arg allele and/or ArgArg genotype (Table 4).

### Median time to progression of *XRCC1* genetic polymorphisms

Four studies including 349 advanced lung cancer patients were finally included in this part of analysis for the *XRCC1* Arg399Gln polymorphism. Because the data from these enrolled studies on the association of *XRCC1* Arg399Gln polymorphism with median TTP was too insufficient to be conducted meta-analysis, we only summarized the general predictive value of the median TTP for *XRCC1* Arg399Gln polymorphism in advanced lung cancer patients. The lung cancer patients bearing the unfavorable 399Gln allele, GlnGln, and GlnArg genotypes were more likely to lower TTP to platinum-based chemotherapy compared to those with the favorable 399Arg allele, and ArgArg genotype (Table 2).

### Median progression-free survival of *XRCC1* genetic polymorphisms

Three studies with a total number of 721 advanced lung cancer patients were enrolled in the final analysis of the association between *XRCC1* Arg194Trp polymorphism and median PFS. The *XRCC1* Arg194Trp polymorphism was not significantly associated with the median PFS of advanced lung cancer patients treated with platinum-based chemotherapy (TrpTrp vs. ArgArg: HR = 1.01, 95% CI, 0.69–1.48, P = 0.948, Fig. 2C; TrpArg vs. ArgArg: HR = 1.06, 95% CI, 0.82–1.36, P = 0.662; TrpTrp+TrpArg vs. ArgArg: HR = 1.05, 95% CI, 0.79–1.38, P = 0.753, Table 3).

There were six studies, altogether 1056 advanced lung cancer patients, finally qualified for the analysis of the association between *XRCC1* Arg399Gln polymorphism and median PFS. There was no statistically significant evidence for an influence of the *XRCC1* Arg399Gln polymorphism on median PFS of advanced lung cancer patients treated with platinum-based chemotherapy (GlnGln vs. ArgArg: HR = 0.80, 95% CI, 0.58–1.11, P = 0.179, Fig. 3C; GlnArg vs. ArgArg: HR = 0.91, 95% CI, 0.71–1.17, P = 0.468; GlnGln+GlnArg vs. ArgArg: HR, = 0.86, 95% CI, 0.70–1.06, P = 1.38, Table 4). Stratified analyses by ethnicity indicated the Arg399Gln polymorphism was not statistically significantly associated with median PFS of advanced lung cancer patients in Asians and Caucasians (Table 4).

No other significant differences were detected with respect to the associations between predictive assessment in pharmacogenetics of *XRCC1* genetic polymorphisms and the clinical outcomes of advanced lung cancer patients treated with platinum-based chemotherapy (Tables 3 and 4).

### Publication Bias and sensitivity analysis

The publication bias in the enrolled studies on the association of the *XRCC1* Arg194Trp and Arg399Gln polymorphisms with clinical outcomes of advanced lung cancer patients treated with platinum-based chemotherapy was assessed by the Begg’s funnel plot and Egger’s test. As shown in Fig. S1, the shapes of the Begg’s funnel plots under the homozygote comparison model of Arg194Trp polymorphism (TrpTrp vs. ArgArg) shown approximately symmetrical, and significant evidence of publication bias was not observed by the Egger’s test. As for the Arg399Gln polymorphism, the shapes of the Begg’s funnel plots under the heterozygote comparison model (GlnArg vs. ArgArg) seemed approximately symmetrical, while the Egger’s test show potentially evidence of publication bias (Fig. S2). Sensitivity analysis showed that changing the effect models had no significant effects on the pooled OR and HR, and did not change the final strength of the association between the *XRCC1* Arg194Trp and Arg399Gln polymorphisms and sensitivity to platinum-based chemotherapy for advanced lung cancer patients. The integrated effects of the exclusion of low quality studies were also evaluated, and the results indicated that excluding of low quality studies did not significantly change the final effect, suggesting that the assessment results of this system are reliable.

### Cumulative meta-analyses

**Figure 4:**
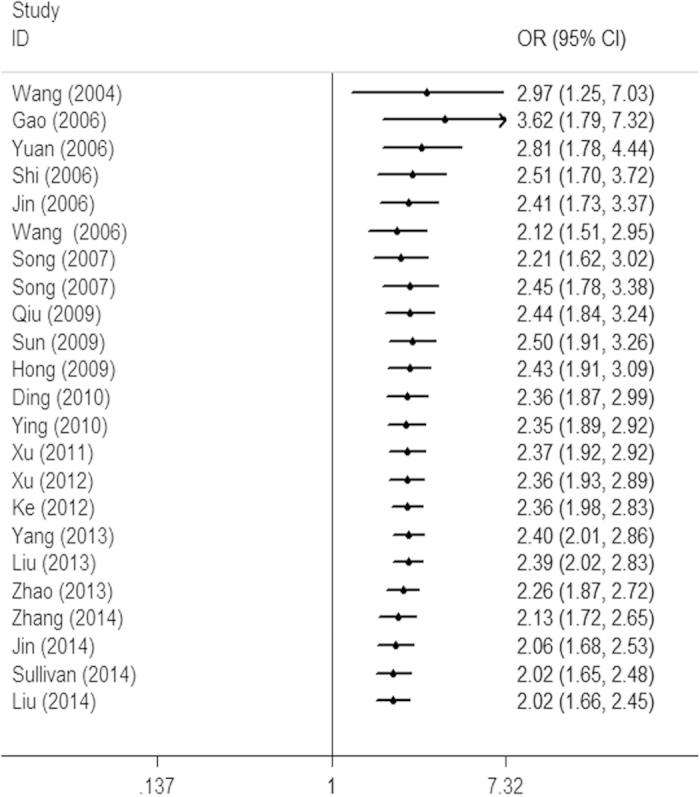
Forest plot of cumulative meta-analysis to sort out the time-tendency of clinical outcomes in advanced lung cancer patients treated with platinum-based chemotherapy by the *XRCC1* Arg194Trp genetic polymorphism (Odds ratios (ORs) and its 95% confidence interval (CI) of objective response rate (ORR) for TrpTrp+TrpArg vs. ArgArg).

**Figure 5:**
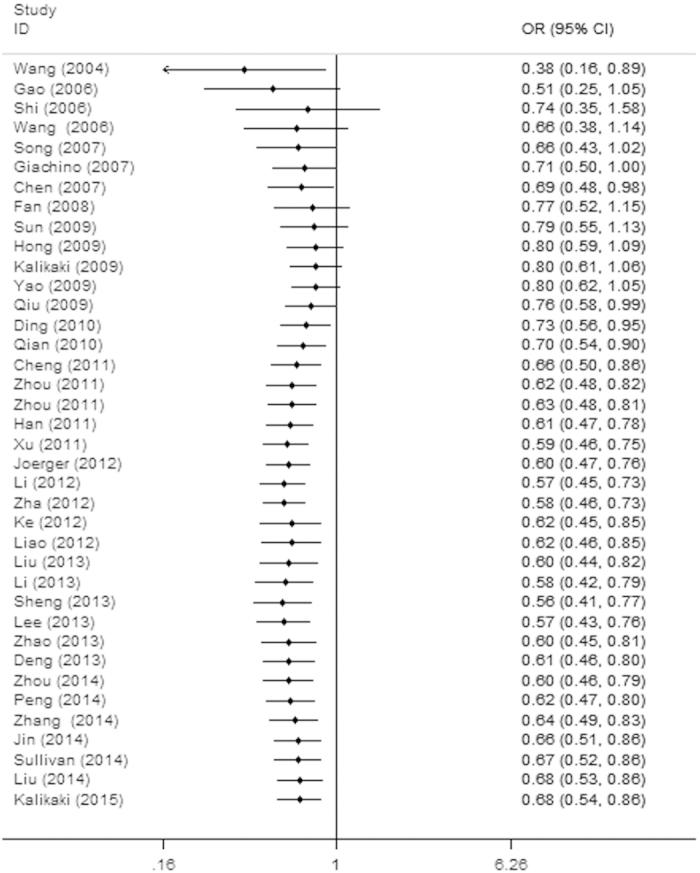
Forest plot of cumulative meta-analysis to sort out the time-tendency of clinical outcomes in advanced lung cancer patients treated with platinum-based chemotherapy by the *XRCC1* Arg399Gln genetic polymorphism (Odds ratios (ORs) and its 95% confidence interval (CI) of objective response rate (ORR) stratified by ethnicity for GlnGln+GlnArg vs. ArgArg).

As shown in Fig. 4, our cumulative meta-analyses based on year of publication indicated that there was a distinct trend toward a better response rate to platinum-based chemotherapy treatment with advanced lung cancer patients for the *XRCC1* Arg194Trp polymorphism (TrpTrp+TrpArg vs. ArgArg, Fig. 4). Between 2004 and 2014, a total of 23 studies were published, with a cumulative effect estimate of OR being 2.02 (95% CI, 1.66–2.45). As for the *XRCC1* Arg399Gln polymorphism, a cumulative meta-analysis of total 38 studies was conducted to evaluate the cumulative effect estimate over time. We found an apparent poorer response rate in advanced lung cancer patients treated with platinum-based chemotherapy (GlnGln+GlnArg vs. ArgArg), especially in the recent studies published for the past 4 to 5 years (Fig. 5). In 2004, Wang *et al*. reported an effect estimate of OR being 0.38 (95% CI, 0.16–0.89). Between 2006 and 2009, a total of 12 studies were published, with a cumulative effect estimate of OR being 0.76 (95% CI, 0.58–0.99). Between 2010 and 2015, a total of 25 more publications were added cumulatively, resulting in an overall effect estimate of OR being 0.68 (95% CI, 0.54–0.86, Fig. 5).

## Discussion

In this study, we found, by an extensively quantitative and systematic review of all available published studies, that patients carrying *XRCC1* 194Trp allele were more likely to better response rates compared to those carrying the 194Arg allele, and patients with *XRCC1* 399Gln allele carries (GlnGln+GlnArg) may be indicative of poorer response rates, shorter survival time and higher risks of death than the 399ArgArg genotype. Thus, we suggested that the *XRCC1* Arg194Trp and Arg399Gln genetic polymorphisms may be predictive factors for treatment response to advanced lung cancer patients treated with platinum-based chemotherapy.

The platinum-based chemotherapy regimens are the standard first-line therapies for advanced lung cancers, and commonly used today. The platinum is an effective chemotherapeutic drug for lung cancer patients. The platinum-based compounds could damage DNA by activating intracellularly to form reactive platinum complexes that covalently bind to DNA to induce intrastrand and interstrand DNA cross-links, as well as DNA-protein cross-links, thereby causing the eventual death of cancer cells^82^. The cancer cells may be more likely to resist against the platinum-based chemotherapy when its ability to repair DNA damage is enhanced by removing those DNA adducts caused by platinum-based compounds^14^,^83^. Previous studies revealed that lung cancer patients carrying a lower DNA repair capacity had an increased OS after the first-line platinum-based chemotherapy^11^,^12^. However, the anti-cancer drug therapeutic efficacy of platinum-based chemotherapy varies largely among different individuals. Genetic polymorphisms of drug target genes, genes involving in detoxification pathways and DNA repair pathways may influence the anti-cancer therapeutic efficacy of platinum drugs and reveal platinum sensitivity in patients^84^,^85^. These genetic polymorphisms could contribute to the variety in phenotypic drug sensitivity through modifying functions of its related genes^14^.

The XRCC1 protein, a limiting factor in the BER pathway, is considered to play key roles in DNA damage repair, and the association of *XRCC1* genetic polymorphisms with sensitivity to platinum-based chemotherapy treatment has attracted much interest and became a research hotpot in individual treatment for lung cancer patients. The *XRCC1* genetic polymorphisms have been proved to be associated with an altered DNA repair activity^11^,^12^. The *XRCC1* transcript abundance levels have shown a significant correlation with DDP chemoresistance in NSCLC cell lines^86^. Published data have indicated that the *XRCC1* genetic polymorphisms might be associated with the clinical outcome of platinum-based chemotherapy treatment in lung cancer patients^7^,^10^,^34^,^37^,^43^,^45^,^54^,^59^,^60^,^63^,^64^,^69^,^71^,^75^,^76^,^87^. These studies imply that the *XRCC1* genetic polymorphisms contribute to the repair of DNA damage induced by the platinum agents. Previous studies have also reported that the *XRCC1* genetic polymorphisms could be potential risk factors for the pathogenesis of lung cancer^88^,^89^,^90^,^91^,^92^ and acting as promising predictive or prognostic biomakers for patients with lung cancer^10^,^14^,^93^,^94^. Thus, it is speculated that the functional SNPs in *XRCC1* gene might be associated with sensitivity to platinum-based anticancer drugs and have predictive or prognostic values in clinical outcomes for patients with lung cancer. The Arg194Trp and Arg399Gln genetic polymorphisms are the most extensively studied SNPs of *XRCC1* gene. These polymorphisms, that lead to the encoded amino acid changes (for Arg194Trp, C→T, Arg→Trp, exon 6; for Arg399Gln, G→A, Arg→Gln, exon 10) which might affect the normal function of XRCC1 protein, might alter the efficiency of DNA repair^14^,^79^,^95^. These polymorphisms are located within the functional domain and could have a significant influence on *XRCC1* function. Although, the Arg280His genetic polymorphism is also an amino acid variant and leads to the Arg to His substitution (G→A, exon 6), the codon 280 is located outside the known functional domains of *XRCC1*^96^. In our study, we also found that few studies have reported the efficacy of clinical outcomes of advanced lung cancer patients with platinum-based chemotherapy treatment for the Arg280His genetic polymorphism. Thus, the study for Arg280His genetic polymorphism was too few to be analyzed in this meta-analysis.

With a pooled dataset of 7433 advanced lung cancer patients treated with platinum-based chemotherapy, we performed a comprehensive and systematic evaluation of clinical outcomes by ORR, OS, TTP, and PFS. We are delighted to find that the *XRCC1* Arg194Trp and Arg399Gln polymorphisms are significantly associated with clinical outcomes in advanced lung cancer patients treated with platinum-based chemotherapy. Our meta-analysis suggested that there were significant associations between the Arg194Trp polymorphism and ORR under all the genetic models; however, there were significant associations between the Arg399Gln polymorphism and ORR only in the dominant genetic model and in the Asian population. It seemed that the Arg194Trp may be a reliable predictive locus to assess the pharmacogenetics of *XRCC1* gene on clinical outcomes of advanced lung cancer patients treated with platinum-based chemotherapy compared with the Arg399Gln based on ORR outcomes. We found that all patients in the enrolled studies examining the Arg194Trp genotypes were Asians, likely because this genetic polymorphism is rare in Caucasians (<6%)^16^,^95^. Besides, most of patients in the enrolled studies examining the Arg399Gln genotypes were Asians, and patients in only seven studies were Caucasians. Lung cancer is a heterogeneous and complicated disease, therefore ethnic difference may affect anti-cancer therapeutic efficacy of platinum drugs. To clarify this concern, we evaluated the relationship of genetic polymorphisms with the anti-cancer therapeutic efficacy of platinum drugs stratified by different ethnic populations. Our results indicated that most of the separately analyzed results were consistent with the overall populations, while the Arg399Gln genetic polymorphism was only significantly associated with ORR in Asian population in the dominant genetic model, not in Caucasian population (Fig. 3A, Table 4). We also noted different clinical outcomes with respect to the Arg399Gln genetic polymorphism (Table 4). These findings show that the clinical outcomes of platinum-based chemotherapy treatment for advanced lung cancer patients differ between Asian and Caucasian populations. Therefore, the conclusions drawn from this meta-analysis about the ethnicity subgroup should be weighed with caution, and the ethnic factor should be considered if individual chemotherapy treatment for lung cancer patients is conducted in the future. The consistent quality of studies or trials is an important influence factor and might vary in meta-analyses of genetic epidemiology studies^79^,^97^,^98^. Wu *et al.* firstly designed the quality assessment system^79^. Here, based on their quality assessment system, we added more clinical variables, such as MST and median PFS. The consistent study quality could be evaluated using the criteria established in this meta-analysis. These criteria, including phenotypic, genetic epidemiologic, and clinical variables, will help standardize study design and experimentation, and might influence future pharmacogenomic studies in the field of lung cancer research. Thus, subgroup analyses were also conducted with respect to QS (Tables 3 and 4). Our results showed that most of the separately analyzed results were consistent with the overall QS. It was noted that the correlation between Arg399Gln genetic polymorphism and clinical outcomes appeared in the low-quality studies, but not in the high-quality studies (Table 4). Results from this study suggested that the predictive role of genetic polymorphisms in clinical outcomes might need to be explored more carefully in future pharmacogenomic studies incorporating more credible criteria in the design and experimentation to obtain more accurate and robust conclusion. We observed that the Arg194Trp and Arg399Gln genetic polymorphisms in *XRCC1* gene were not statistically influenced the median PFS in all advanced lung cancer patients with platinum-based chemotherapy treatment (Figs. 2C and 3C, Tables 3 and 4). These results have not shown significance because there are few studies provided enough median PFS data and finally involved into our meta-analysis. 

To our knowledge, for the first time, we conducted a sequential year-to-year cumulative meta-analysis for clinical outcomes of platinum-based chemotherapy treatment with advanced lung cancer patients. Provided time span of all the available studies was considerable (from 2004 to 2015), our cumulative meta-analysis was encouraged to sort out the cumulative evidence from time-tendency of clinical outcomes by successively adding published studies to the given results. As for the *XRCC1* Arg194Trp polymorphism, sequential cumulative meta-analyses consistently and stably showed equivalent effects of better response rates to platinum-based chemotherapy treatment with advanced lung cancer patients since the several initial studies were pooled, which also showed the stable time-dependent trend (Fig. 4). As for the *XRCC1* Arg399Gln polymorphism, sequential cumulative meta-analyses results were calculated for each year from 2004. Our cumulative meta-analysis did not show a significant change in trend of reporting response rate to platinum-based chemotherapy treatment with advanced lung cancer patients between 2004 and 2009, which range from 0.38 to 0.76. Nonetheless, from 2010 onwards, it is clear from the sequential pooled cumulative meta-analyses results from studies that there is consistently and stably showed an apparent trend toward a poorer response rate to platinum-based chemotherapy treatment with advanced lung cancer patients, which range from 0.73 to 0.68 (Fig. 5). The trial sequential analysis of cumulative meta-analyses (performing a new meta-analysis each time the results of a new clinical trial are published) would have made the evidence much clearer earlier, and lead to sufficient assessment of clinical outcomes. It may also have prevented many redundant trials, redirected trial design, and help planning further clinical trials. Therefore, the conclusions drawn from this cumulative meta-analysis about the response rates to platinum-based chemotherapy treatment with advanced lung cancer patients should be weighed with caution.

Several previous published meta-analyses have been carried out to reveal the potential correlation of *XRCC1* gene polymorphisms and platinum-based chemotherapy in lung cancer patients, these results remain conflicting rather than conclusive^14^,^15^,^16^,^79^,^80^,^81^. The different studies which enrolled in the meta-analysis could possibly biase the conclusions. In the current meta-analysis, we systematically summarized all available up-to-date studies on the association of *XRCC1* gene polymorphisms with platinum-based chemotherapy treatment for lung cancer through conducting to comprehensive literature search in multiple databases without limiting publication date and language. As a result, our updated meta-analysis enrolled 53 available studies including 7433 advanced lung cancer patients, which were significantly more than the previous published meta-analyses. Therefore, findings from this study could provide more precise and reliable comprehensive assessment than those published meta-analyses on the predictive role of *XRCC1* genetic polymorphisms in clinical outcomes of platinum-based chemotherapy treatment for advanced lung cancer patients. 

Despite our efforts in performing a comprehensive analysis, several limitations of this meta-analysis should be taken into consideration when interpreting the present results. Firstly, the sample sizes of enrolled studies were from 45 to 460, and the small sample size of studies might not generate solid conclusion in some situation. Some of the findings in subgroups may have been undervalued because of there was only one trial available for analyses. Secondly, the significant heterogeneity between studies was found in our pooled analysis, while it is unlikely to alter our main conclusions because our results reflected the most current state of this issue in clinical practice and studies. Stratified analyses by possible confounding factors that could contribute to the ORR and OS of patients, such as gender, age, ethnicities, cancer pathology types, cancer stage, smoking history, specific anti-cancer drugs, chemotherapy regimens, test methods, and toxic effects of various platinum-based therapies or other chemotherapies, could provide additional useful information and be helpful to reduce the heterogeneity. However, few of these studies provided sufficient data by subgroups, thus making such subgroup analyses were impossible to implement in the present study. Furthermore, our analyses mostly used unadjusted estimates, because not all published studies calculate adjusted estimates by possible confounding factors. A more precise analysis with the adjustment estimates could be conducted if more detailed individual data were available. Therefore, in order to make the result more accurate and reliable, future studies are necessary to avoid these pitfalls. Thirdly, among all 53 trials utilized in this meta-analysis, most of studies were conducted in Asians (Chinese and Korean populations), only seven studies in Caucasian populations (Greek, Spanish, Swiss, Italian and American populations). Thus, the findings from this meta-analysis might be applicable only to these ethnic populations, while could limit the generalizability to other patient populations. Fourthly, only published studies in English and Chinese were enrolled in this meta-analysis, published studies in other languages, ongoing studies and unpublished data were not collected, which could cause some biases in our estimates.

## Conclusions

In conclusion, despite the limitations, our meta-analysis indicates a predictive role for the genetic polymorphisms of *XRCC1* gene in clinical outcomes of platinum-based chemotherapy for advanced lung cancer patients. The *XRCC1* Arg194Trp and Arg399Gln polymorphisms are likely to be associated with the ORR, OS and sensitivity to platinum-based chemotherapy for advanced lung cancer patients. The use of *XRCC1* gene polymorphisms as predictive factor of clinical outcomes in personalized chemotherapy treatment for lung cancer patients might need to be investigated more carefully in well-designed pharmacogenetics and functional studies with large sample sizes in diverse ethnic populations to ensure a more accurate and robust conclusion in the future.

## Methods

### Identification of eligible studies

We conducted a systematic literature search using the following search terms “lung cancer or tumour or tumor or neoplasm or carcinoma”, “*XRCC1* or X-ray repair cross complementing group 1”, “base excision repair” or “BER”, “SNPs or genetic polymorphisms or variations”, “pharmacogenomics”, “platinum or cisplatin or carboplatin or nedaplatin, lbaplatin, oxaliplatin” and “chemotherapy” from PubMed, MEDINE, Excerpta Medica Database (EMBASE), ISI Web of Science, ScienceDirect, Wiley Online Library, Cochrane Central Register of Controlled Trials, Chinese Biomedical Literature Database (CBM), Wangfang Data, and Chinese National Knowledge Infrastructure (CNKI) databases up to March 30, 2015. No restriction of publication date was applied. Reference lists were screened manually to further identify additional eligible studies. We retrieved all potentially studies to identify the most eligible literatures.

### Inclusion and exclusion criteria

The following inclusion criteria for the current meta-analysis were as follows: (1). Patients had pathologically or histologically confirmed advanced lung cancer. (2). *XRCC1* genetic polymorphisms were genotyped; (3). Treatments had platinum-based chemotherapy; (4). Studies had sufficient data to estimate relative risks for prognostic effects of advanced lung cancer (i.e., ORR, OS, median PFS, MST, TTP, OR and HR with corresponding to 95% CIs); (4). only full-text studies were included. Studies were excluded if any of the following exclusion criteria applies: (1). Duplicated publications; (2). Abstracts, comments, letters, and review articles; (3). Not reported any clinical outcomes; (4). No sufficient data were reported. (5). The studies with animals or cell lines were reported; (6). The corresponding authors were not provided the valid data or critical information upon our request.

### Data extraction

Two investigators (Z.R. Yuan and J. Li) independently extracted data from each eligible publications. Discrepancies between investigators were resolved by discussion from the third investigator, or the team’s decision. The following information was collected from the included studies: the first author’s name, year of publication, country, ethnicities (categorized as Asian and Caucasian), number of patients, median age (year), clinical stage, evaluation criterion, genotyping methods, outcomes (ORR, OS, PFS, MST, TTP, with corresponding to 95% CI), and the number of responders and non-responders in different genotypes.

### Quality assessment

The QS of each eligible literature was also evaluated separately by two investigators (Z.R. Yuan and J. Li) using a predefined scale (Table 5). Based on the previous published studies^79^,^81^,^97^,^99^,^100^, the scale for quality assessment and quality scoring criteria were determined with the following eight factors: cancer clinical stage, evaluation criteria, platinum combinations, genotyping methods, OS, median PFS, MST, and sample size (Table 5). The total QS ranged from 0 (worst) to 24(best). The final QS were assigned to each included literatures after disagreement discussed and resolved by consensus from reviewers. The literature with QS ≤ 12 (or >12) was considered low (or high) quality.

### Statistical analysis

Five genetic models, including homozygote genetic model (mutational homozygote vs. wild homozygote), heterozygote comparison (heterozygote vs. wild homozygote), dominant genetic model (heterozygote+mutational homozygote vs. wild homozygote), recessive genetic model (mutational homozygote vs. heterozygote+wild homozygote), and allele genetic model (mutational vs. wild) were considered in this meta-analysis. We extracted the ORR, OS, ORs, HRs, MST, PFS, and TTP from all enrolled studies. We evaluated the ORs and 95% CIs for the ORR and no response after platinum-based chemotherapy (complete response (CR) + partial response (PR) vs. progressive disease (PD) + stable disease (SD), using the World Health Organization (WHO) or the Response Evaluation Criteria in Solid Tumors (RECIST) criteria). We conducted the PRISMA checklist as the protocol of this meta-analysis and followed its guideline (Fig. S3 and Table S1)^101^. The strength of association between *XRCC1* genetic polymorphisms and response rate of platinum-based chemotherapy for advanced lung cancer patients was estimated by calculating pooled ORs with corresponding to 95% CIs. The significance of the pooled ORs was estimated using the Z-test, and P-value < 0.05 was considered statistically significant. HRs and 95% CIs were estimated for OS, MST, median TTP, median PFS, directly from the raw data of enrolled articles, or indirectly from the Kaplan–Meier (KM) curve of enrolled articles using published methods^102^,^103^. Subgroup analyses were detected by stratified patient populations, clinical outcomes, and QS. The between-study heterogeneity was evaluated by the Cochran’s chi-square-based Q-test^104^,^105^ and the I^2^ index^106^. P-value < 0.10 and/or I^2^ index >50% for Q-test indicated the existence of heterogeneity between studies^107^, so the pooled ORs was calculated by the random-effects model using the DerSimonian and Laird method^108^. Otherwise, the fixed-effects model (the Mantel-Haenszel method) was used^109^. The sensitivity was conducted by changing the effect models and excluding low quality studies (defined as QS ≤ 12) to estimate confidence. The effects of publication bias of literatures were examined by the Begg’s funnel plots^110^, and Egger’s linear regression method^111^. Cumulative meta-analyses were performed to sort out the time-tendency of clinical outcomes of platinum-based chemotherapy for advanced lung cancer patients. All tests were two-sided and P-values < 0.05 were considered representative of statistically significant level. All analyses were conducted using the STATA software (version 11.0; STATA Corporation, College Station, TX, USA).

## Additional Information

**How to cite this article**: Yuan, Z. *et al.* Predictive assessment in pharmacogenetics of *XRCC1* gene on clinical outcomes of advanced lung cancer patients treated with platinum-based chemotherapy. *Sci. Rep.* **5**, 16482; doi: 10.1038/srep16482 (2015).

## Supplementary Material

Supplementary Information

## Figures and Tables

**Table 1:** The general characteristics of eligible studies in the meta-analysis.

Study	Year	Country	Ethnicity	Numberofpatients	Medianage(year)	Clinicalstage	Evaluationcriterion	Outcomes	Genotypingmethods	Arg194Trp genotypedistribution			Arg399Gln genotypedistribution			HR	QS
										Trp/Trp	Trp/Arg	Arg/Arg	Gln/Gln	Gln/Arg	Arg/Arg		
Wang *et al.*,^29^	2004	China	Asian	105	56(30–74)	IIIB-IV	WHO	ORR	PCR-RFLP	3/11^a^	19/18^a^	11/43^a^	2/8^a^	9/33^a^	22/31^a^	NR	13
Gurubhagavatula *et al.*,^10^	2004	USA	Caucasian	103	58(32–77)	IIIA/B-IV	RECIST	OS/MST	PCR-RFLP	—	—	—	10^b^	42^b^	51^b^	HR	19
Gao *et al.*,^30^	2006	China	Asian	57	59(38–77)	II-IV	WHO	ORR	PCR-RFLP	2/2^a^	12/11^a^	5/25^a^	0/3^a^	8/15^a^	11/20^a^	NR	12
Yuan *et al.*,^31^	2006	China	Asian	200	56(30–74)	IIIB-IV	WHO	ORR	PCR-RFLP	10/13^a^	38/46^a^	24/69^a^	—	—	—	NR	14
Shi *et al.*,^32^	2006	China	Asian	112	60(22–81)	II-IV	WHO	ORR	PCR-RFLP	9/3^a^	24/27^a^	18/30^a^	4/7^a^	19/17^a^	28/37^a^	NR	13
Jin *et al.*,^33^	2006	China	Asian	162	(28–75)	IIIB-IV	WHO	ORR	PCR-RFLP	10/14^a^	35/25^a^	27/51^a^	—	—	—	NR	14
de las Penas R. *et al.*,^34^	2006	Spain	Caucasian	135	62(31–81)	IIIB-IV	RECIST	OS/MST	TaqMan	—	—	—	18^b^	63^b^	49^b^	HR	20
Wang *et al.* ,^35^	2006	China	Asian	135	55(29–74)	IIIB-IV	WHO	ORR/MST	PCR-RFLP	34/35^a^^,^^c^		30/36^a^	25/40^a^^,^^d^		39/31^a^	NR	16
Song *et al.*,^36^	2007	China	Asian	97	56(30–68)	IIIB-IV	WHO	ORR	PCR-RFLP	3/8^a^	19/22^a^	8/37^a^	1/4^a^	11/29^a^	18/34^a^	NR	12
Giachino *et al.*^37^	2007	Italy	Caucasian	248	62(41–79)	IIIA/B-IV	RECIST	ORR/OS/MST	PCR-RFLP	—	—	—	12/17^a^	18/82^a^	31/88^a^	HR	17
Song *et al.*,^38^	2007	China	Asian	166	56(30–68)	IIIB-IV	WHO	ORR	PCR-RFLP	4/12^a^	34/32^a^	14/70^a^	—	—	—	NR	14
Chen *et al.*, 39	2007	China	Asian	64	55(20–75)	Advanced	RECIST	ORR/PFS	TaqMan	—	—	—	0/2^a^	20/40^a^	2/0^a^	NR	10
Liu *et al.*,^40^	2008	China	Asian	53	61(28–74)	I-IV	RECIST	OS/MST/TTP	TaqMan	—	—	—	8^b^	18^b^	27^b^	HR	19
Fan *et al.*,^41^	2008	China	Asian	81	62.9(55–80)	IIIB–IV	WHO	ORR	PCR-RFLP	—	—	—	16/20^a^^,^^d^		13/32^a^	NR	**12**
Qiu *et al.*,^42^	2009	China	Asian	107	NR	III-IV	WHO	ORR	PCR-RFLP	7/6^a^	27/23^a^	14/30^a^	—	—	—	NR	13
Sun *et al.*,^43^	2009	China	Asian	87	59(34–79)	IV	WHO	ORR	3D DNA microarray	5/6^a^	18/19^a^	8/31^a^	1/3^a^	8/22^a^	14/39^a^	NR	13
Hong *et al.*, 44	2009	China	Asian	164	61(27–84)	III-IV	WHO	ORR	PCR-RFLP	7/11^a^	31/42^a^	19/54^a^	3/10^a^	28/53^a^	26/44^a^	NR	14
Kalikaki *et al.*,^45^	2009	Greece	Caucasian	119	61(39–85)	IIIA/B-IV	RECIST	ORR/OS/MST	PCR-RFLP	—	—	—	26/60^a,d^		11/21^a^	HR	17
Yao *et al.*,^46^	2009	China	Asian	108	61(39–79)	IIIA/B-IV	WHO	ORR/OS/MST	PCR-RFLP	—	—	—	9/48^a^, 60^b^	12/28^a^, 43^b^	1/4^a^, 5^b^	HR	19
Qiu *et al.*,^47^	2009	China	Asian	107	NR	III-IV	RECIST	ORR	PCR-RFLP	—	—	—	2/6^a^	14/26^a^	32/27^a^	NR	13
Ding *et al.*,^48^	2010	China	Asian	54	60(40–85)	IIIB-IV	WHO	ORR	DNA Sequencing	4/3^a^	9/10^a^	12/16^a^	3/10^a^	4/6^a^	18/13^a^	NR	13
Qian *et al.*,^49^	2010	China	Asian	107	NR	IIIB-IV	WHO	ORR	PCR-RFLP	—	—	—	2/6^a^	14/26^a^	32/27^a^	NR	13
Yuan *et al.*,^6^	2010	China	Asian	199	56(29–74)	IIIA-IV	WHO	OS/PFS/MST	PCR-RFLP	23^b^	83^b^	93^b^	20^b^	74^b^	105^b^	HR	23
Ying *et al.*,^50^	2010	China	Asian	80	30–78	IIIB-IV	WHO	ORR	PCR-RFLP	5/7^a^	17/12^a^	13/26^a^	—	—	—	NR	12
Cheng *et al.*^51^,^52^	2011	China	Asian	120	58(34–77)	Advanced	WHO	ORR/MST	DNA microarray	—	—	—	5/14^a^	9/44^a^	21/27^a^	KM	15
Han *et al.*,^53^	2011	Korea	Asian	158	57(19–74)	IIIB-IV	NR	OS/PFS	TaqMan	—	—	—	8^b^	63^b^	87^b^	HR	12
Zhou *et al.*,^54^	2011	China	Asian	111	57(42–71)	IV	RECIST	ORR/TTP	DNA Sequencing	—	—	—	6/34^a^^,^^d^		29/42^a^	NR	14
Zhou *et al.*, 55	2011	China	Asian	94	57(42–71)	IIIB–IV	RECIST	ORR/TTP	DNA Sequencing	—	—	—	11/31^a^^,^^d^		19/33^a^	KM	14
Han *et al.*,^56^	2011	China	Asian	91	56	IV	RECIST	ORR/TTP	DNA Sequencing	—	—	—	8/33^a^^,^^d^		20/30^a^	NR	10
Xu *et al.*,^57^	2011	China	Asian	130	62(28–83)	IIIB-IV	RECIST	ORR	PCR-RFLP	18/18^a^	14/26^a^	12/42^a^	0/10^a^	14/40^a^	30/36^a^	NR	13
Hong *et al.*,^58^	2011	China	Asian	262	NR	I-IV	NR	OS/MST	TaqMan	—	—	—	20^b^	77^b^	165^b^	HR	13
Joerger *et al.*,^59^	2012	Switzerland	Caucasian	131	59.7(37–79)	IIIB–IV	RECIST	ORR/OS/PFS/MST	DNA Sequencing	—	—	—	5/12^a^	18/45^a^	17/34^a^	HR	23
Li *et al.*,^60^	2012	China	Asian	89	59.08(21–84)	III–IV	RECIST	ORR	DNA Sequencing	—	—	—	6/39^a^^,^^d^		20/24^a^	NR	13
Xu *et al.*,^61^	2012	China	Asian	149	62(28–83)	IIIB-IV	RECIST	ORR	PCR-RFLP	9/9^a^	24/38^a^	16/53^a^	—	—	—	NR	13
Zha *et al.*,^62^	2012	China	Asian	52	63(45–75)	IIIA-IIIB	WHO	ORR	PCR-LDR	—	—	—	13/15^a^^,^^d^		13/11^a^	NR	12
Ke *et al.*,^63^	2012	China	Asian	460	55(32–79)	I-IV	NR	ORR/OS	PCR-CTPP	44/19^a^	45/52^a^	104/196^a^	36/15^a^	85/92^a^	72/160^a^	HR	14
Liao *et al.*,^64^	2012	China	Asian	62	57(36–78)	IIIB-IV	NR	ORR/OS/PFS/MST	SNPstream UHT	—	—	—	1/4	9/22	9/17	HR	19
Liao *et al.*,^64^	2012	China	Asian	45	63(43–83)	IIIB-IV	NR	OS/PFS/MST	SNPstream UHT	—	—	—	2^b^	24^b^	19^b^	HR	16
Liu *et al.*,^65^	2013	China	Asian	62	58(37–72)	Advanced	RECIST	ORR	TaqMan	—	—	—	4/23^a^		15/20^a^	NR	10
Li *et al.*,^66^	2013	China	Asian	83	63.07	IIIA-IV	WHO	ORR	PCR-RFLP	—	—	—	1/5^a^	3/25^a^	21/28^a^	NR	9
Yang *et al.*,^67^	2013	China	Asian	54	56(30–73)	IIIB–IV	RECIST	ORR/MST	PCR-RFLP	3/1^a^	10/4^a^	13/23^a^	—	—	—	NR	15
Sheng *et al.*,^68^	2013	China	Asian	62	58(37–72)	Advanced	RECIST	ORR	TaqMan	—	—	—	1/4^a^	3/19^a^	15/20^a^	NR	10
Lee *et al.*,^69^	2013	Korea	Asian	382	NR	III-IV	NR	ORR/OS/MST	Sequenome MS-based genotyping assay	—	—	—	5/16^a^	64/75^a^	110/100^a^	HR	18
Liu *et al.*,^70^	2013	China	Asian	200	56(30–74)	IIIB-IV	NR	ORR	PCR-RFLP	10/13^a^	38/46^a^	24/69^a^	—	—	—	NR	11
Zhao *et al.*,^71^	2013	China	Asian	147	60(32–82)	IIIB-IV	RECIST	ORR/OS/PFS/MST	TaqMan	1/6^a^	20/35^a^	32/51^a^	8/5^a^	24/31^a^	21/56^a^	HR	23
Deng *et al.*,^72^	2013	China	Asian	97	57(31–79)	IIIB-IV	RECIST	ORR/PFS	PCR-RFLP and DNA Sequencing	—	—	—	9/35^a^^,^^d^		16/37^a^	NR	15
Zhou *et al.*, 73	2014	China	Asian	204	61(45–75)	NR	RECIST	ORR/OS/MST	MALDI-TOF-MS	—	—	—	23/78^a^^,^^d^		38/65^a^	KM	16
Peng *et al.*,^7^	2014	China	Asian	235	58(29–84)	IIIA-IV	RECIST	ORR/OS/PFS/MST	PCR-CTTP	—	—	—	3/6^a^	41/74^a^	40/71^a^	HR	20
Zhang *et al.*,^74^	2014	China	Asian	375	60.9	IIIA-IV	NR	ORR/OS/PFS/MST	MassARRAY	23/41^a^	44/90^a^	60/118^a^	24/29^a^	54/94^a^	49/125^a^	HR	21
Jin *et al.*,^75^	2014	China	Asian	378	62.4(36–78)	I-IV	NR	ORR/OS/DFS	PCR-RFLP	25/29^a^	48/71^a^	71/134^a^	28/19^a^	64/96^a^	52/119^a^	HR	14
Sullivan *et al.*,^76^	2014	Spain	Caucasian	161	63.7(36–85)	IIIA-IV	RECIST	ORR/OS/PFS	TaqMan	11/8^a^^,^^c^	78/64^a^	13/14^a^	39/33^a^		37/25^a^	NR	15
Liu *et al.*,^77^	2014	China	Asian	82	59.85(29–78)	Advanced	NR	ORR	PCR-RFLP	4/5^a^	16/19^a^	11/27^a^	2/6^a^	14/23^a^	13/24^a^	NR	6
Kalikaki *et al.*,^78^	2015	Greece	Caucasian	107	60.0(37–78)	IIIB-IV	RECIST	OS/PFS/MST	PCR-RFLP	—	—	—	23/44^a^^,^^d^		16/22^a^	HR	22

Note: NR: not reported; QS, quality score; HR: hazard ratio; ORR: objective response rate; OS, overall survival (months); KM, Kaplan-Meier curve; DFS, disease-free survival (months); PFS, progression-free survival (months); MST, median survival time (months); TTP, time to progression (months); 3D, 3-dimensional; PCR, polymerase chain reaction; PCR-RFLP, PCR-restriction fragment length polymorphism; RECIST, Response Evaluation Criteria in Solid Tumors; WHO, World Health Organization; MALDI-TOF-MS, matrix-assisted laser desorption/ionization time of light mass spectrometry; PCR-LDR, PCR-ligase detection reaction; PCR-CTPP, duplex PCR with the confronting-two-pair primer; Sequenome MS-based genotyping assay, sequenome mass spectrometry-based genotyping assay; SNPstream UHT, SNPstream ultra high throughput; PCR-CTTP, PCR with confronting two-pair primers.

^a^Number of patients for ORR; in front of oblique line is good responder (complete response (CR) + partial response (PR)) and behind oblique line is poor responder (stable disease (SD) + progressive disease (PD)).

^b^Number of patients for OS.

^c^Number of patients for Trp/Trp and Trp/Arg genotypes.

^d^Number of patients for Gln/Gln and Gln/Arg genotypes.

**Table 2:** Association between the *XRCC1* Arg194Trp and Arg399Gln polymorphisms and median survival time, median time to progression, and median progression-free survival of platinum-based chemotherapy in advanced lung cancer patients.

Study	Year		Arg194Trp(95%-CI)		Trp/Trp+Trp/Arg	ArgArg	Arg399Gln(95%-CI)		Gln/Gln+Gln/Arg	ArgArg
			TrpTrp	TrpArg			GlnGln	GlnArg		
Cheng *et al.*,	2011	MST	—	—	—	—	—	—	11.10	15.20
Giachino *et al.*,	2007	MST	—	—	—	—	18.67	12.74	—	12.97
		HR	—	—	—	—	0.60(0.35–1.03)	1.17(0.85–1.59)	—	1(Reference)
de las Penas R. *et al.*,	2006	MST	—	—	—	—	10.56(5.03–16.09)	13.95(10.92–16.97)	—	10.86(7.40–14.31)
		HR	—	—	—	—	1.59(0.81–3.10)	1(Reference)	—	1.51(1.03–2.40)
Gurubhagavatula *et al.*,	2004	MST	—	—	—	—	7.70	11.40	—	17.30
		HR	—	—	—	—	3.17(1.48–6.77)	1.22(0.76–1.94)	—	1(Reference)
Kalikaki *et al.*,	2009	MST	—	—	—	—	7.10(0.30–13.90)	11.30(8.90–13.80)	—	14.80(9.10–20.50)
		HR	—	—	—	—	4.58(1.92–10.92)	1.43(0.85–2.40)	—	1(Reference)
Liu *et al.*,	2008	MST	—	—	—	—	8.00(5.90–10.10)	16.00(13.90–18.10	—	24.00(16.50–31.50)
		HR	—	—	—	—	6.24(1.86–20.91)	1.44(0.66–3.12)	—	1(Reference)
Han *et al.*,	2011	MST	—	—	—	—	—	—	—	—
		HR	—	—	—	—	—	—	1(Reference)	1.35(0.90–2.00)
Yao *et al.*,	2009	MST	—	—	—	—	15.00(11.90–21.10)	21.00(11.50–30.90)	—	29.00(7.00–39.00)
		HR					1(Reference)	0.83(0.49–1.41)	—	0.58(0.17–2.04)
Yuan *et al.*,	2010	MST	15.00(9.05–20.50)	17.00(14.40–19.90)	17.00(14.60–19.40)	16.00(10.90–21.10)	14.00(5.70–22.30)	16.00(11.40–20.60)	16.00(12.10–19.9)	17.00(13.80–20.20)
		HR	1.23(0.73–2.10)	0.94(0.66–1.34)	1.00(0.71–1.39)	1(Reference)	1.13(0.63–2.03)	1.25(0.88–1.79)	1.23(0.88–1.71)	1(Reference)
Wang *et al.* ,	2006	MST	—	—	13.00	11.00	—	—	10.00	14.00
Joerger *et al.*,	2012	MST	—	—	—	—	6.00(2.30–9.30)	10.40(9.40–13.70)	—	10.80(7.30–15.90)
		HR	—	—	—	—	1(Reference)	0.62(0.34–1.11)	—	0.56(0.30–1.01)
Zhou *et al.*,	2014	MST	—	—	—	—			10.00	12.00
Yang *et al.*,	2013	MST			14.00	10.00	—	—	—	—
Ke *et al.*,	2012	MST	—	—	—	—	—	—	—	—
		HR	0.45(0.23–0.87)	1.23(0.81–1.89)	—	1(Reference)	0.42(0.21–0.82)	0.76(0.53–1.07)	—	1(Reference)
Lee *et al.*,	2013	MST	—	—	—	—	9.80(2.60–17.00)	13.00(11.10–15.00)	—	14.40(12.70–16.10)
		HR	—	—	—	—	1.47(0.91–2.37)	1.13(0.90–1.42)	—	1(Reference)
Liao *et al.*,	2012	MST	—	—	—	—	—	—	—	22.00(10.00–34.00)
		HR	—	—	—	—	0.25(0.03–1.88)	0.26(0.11–0.64)	—	1(Reference)
Liao *et al.*,	2012	MST	—	—	—	—	—	—	45.00(36.00–54.00)	29.00(20.00–38.00)
		HR	—	—	—	—	—	—	0.47(0.25–0.92)	1(Reference)
Zhao *et al.*,	2013	MST	8.50^a^	32.00	—	14.60^a^	5.70^a^	36.00	—	32.00
		HR	1.13(0.30–4.17)	0.84(0.45–1.58)	0.88(0.48–1.61)	1(Reference)	1.32(0.43–4.00)	0.83(0.44–1.57)	0.89(0.49–1.62)	1(Reference)
Hong *et al.*,	2011	MST	—	—	—	—	—	—	15.00(8.55–21.45)	19.00(15.00–23.00)
		HR	—	—	—	—	—	—	1.27(0.93–1.75)	1(Reference)
Peng *et al.*,	2014	MST	—	—	—	—	16.00(0.00–33.53)	12.00(10.03–13.97)	12.00(10.03–13.97)	17.00(14.69–19.31)
		HR	—	—	—	—	—	—	1(Reference)	1.69(1.19–2.40)
Zhang *et al.*,	2014	MST	26.60(14.90–28.80)	25.30(14.40–29.40)	25.90(13.80–29.10)	23.40(14.20–28.50)	27.50(15.80–32.30)	23.70(14.30–27.40)	25.60(15.20–28.20)	22.30(13.50–27.20)
		HR	0.83(0.51–1.62)	0.79(0.55–1.67)	0.81(0.56–1.66)	1(Reference)	0.48(0.25–0.86)	0.74(0.48–1.53)	0.55(0.23–0.94)	1(Reference)
Jin	2014	MST	—	—	—	—	—	—	—	—
		HR	0.82(0.47–1.39)	0.91(0.61–1.33)	—	1(Reference)	0.51(0.26–0.98)	0.87(0.60–1.24)	—	1(Reference)
Kalikaki *et al.*,	2015	MST	—	—	—	—	—	—	10.80(7.30–14.30)	10.80(4.60–17.90)
		HR	—	—	—	—	—	—	1.01(0.64–1.50)	1(Reference)
Liu *et al.*,	2008	Median TTP	—	—	—	—	4.10(2.30–5.90)	6.00(3.10–8.90)	—	11.00(6.40–15.60)
		HR	—	—	—	—	1.91(0.62–5.83)	1.00(0.50–2.04)	—	1(Reference)
Zhou *et al.*,	2011	Median TTP	—	—	—	—	—	—	6.00(5.50–6.50)	8.50(7.86–9.14)
Zhou *et al.*,	2011	Median TTP	—	—	—	—	—	—	6.50(5.90–7.10)	7.00(6.04–7.96)
Han *et al.*,	2011	Median TTP	—	—	—	—	—	—	5.00	9.50
Yuan et al.,	2010	Median PFS	6.80(4.30–9.30)	7.00(6.00–8.00)	6.80(5.60–8.00)	6.90(5.60–8.30)	6.90(2.50–11.30)	6.90(6.00–7.80)	6.90(6.00–7.80)	6.80(5.60–8.00)
		HR	1.31(0.77–2.23)	1.10(0.78–1.57)	1.14(0.82–1.59)	1(Reference)	0.69(0.38–1.25)	1.08(0.76–1.53)	0.98(0.71–1.35)	1(Reference)
Joerger *et al.*,	2012	Median PFS	—	—	—	—	4.80(1.40–7.30)	6.30(5.30–8.10)	—	5.20(3.50–7.60)
		HR	—	—	—	—	1(Reference)	0.91(0.53–1.58)	—	1.03(0.59–1.82)
Liao *et al.*,	2012	Median PFS	—	—	—	—	5.10(3.10–7.20)	5.10(3.30–7.00)	—	5.80(4.20–7.40)
Zhao *et al.*,	2013	Median PFS	6.70^a^	9.00	—	11.50	8.00	12.00	—	10.00
		HR	0.56(0.16–1.95)	1.12(0.70–1.80)	—	1(Reference)	1.33(0.56–3.19)	0.73(0.45–1.19)	—	1(Reference)
Deng *et al.*,	2013	Median PFS	—	—	—	—	—	—	6.07	6.23
		HR	—	—	—	—			0.81(0.51–1.27)	1(Reference)
Peng *et al.*,	2014	Median PFS	—	—	—	—	2.00(1.03–2.97)	6.00(4.21–7.80)	6.00(4.33–7.67)	7.00(5.69–8.31)
Zhang *et al.*,	2014	Median PFS	9.40(4.20–16.40)	8.70(3.80–15.10)	8.80(4.30–16.30)	8.60(3.40–14.20)	10.90(5.40–18.60)	8.50(3.20–14.20)	10.40(5.10–18.30)	7.80(3.20–13.60)
		HR	0.83(0.42–1.43)	0.89(0.54–1.60)	0.85(0.57–1.59)	1(Reference)	0.51(0.23–0.96)	0.79(0.52–1.58)	0.61(0.31–1.22)	1(Reference)
Kalikaki *et al.*,	2015	Median PFS	—	—	—	—	—	—	4.40(3.00–6.10)	5.60(3.20–8.10)
		HR				—	—	—	0.83(0.55-1.26)	1(Reference)

Note: HR: hazard ratio; MST, median survival time (months); TTP, time to progression (months); PFS, progression-free survival (months).

^a^The mean survival time is shown in the reference.

**Table 3:** Meta-analysis of the association between *XRCC1* Arg194Trp polymorphism and platinum-based chemotherapy in objective response rate, overall survival and median progression-free survival for advanced lung cancer patients.

Genetic comparisons	Study groups	No. ofstudies^a^	Test of association			Model	Test of heterogeneity		
			OR^b^/HR^c^ (95% CI)	Z	P-value		χ^2^	P-value	I^2^(%)
Objective response rate(OR)									
TrpTrp vs. ArgArg	Overall	21	2.07(1.67–2.58)	6.54	<0.001	F	21.87	0.348	8.5
	QS								
	High quality	16	2.08(1.66–2.63)	6.13	<0.001	F	20.86	0.141	28.1
	Low quality	5	2.01(1.11–3.66)	2.29	0.022	F	1.00	0.910	0
TrpArg vs. ArgArg	Overall	21	2.11(1.68–2.65)	6.43	<0.001	R	37.21	0.011	46.3
	QS								
	High quality	16	1.96(1.51–2.54)	5.06	<0.001	R	30.81	0.009	51.3
	Low quality	5	2.83(1.90–4.22)	5.10	<0.001	R	2.22	0.696	0
TrpTrp+TrpArg vs. ArgArg	Overall	23	2.02(1.66–2.45)	7.04	<0.001	R	37.83	0.019	41.8
	QS								
	High quality	18	1.91(1.53–2.38)	5.68	<0.001	R	32.61	0.013	47.9
	Low quality	5	2.63(1.80–3.84)	5.00	<0.001	R	2.11	0.715	0
TrpTrp vs. TrpArg+ArgArg	Overall	21	1.56(1.27–1.91)	4.24	<0.001	F	25.52	0.182	21.6
	QS								
	High quality	16	1.62(1.30–2.02)	4.30	<0.001	F	23.68	0.071	36.7
	Low quality	5	1.21(0.69–2.11)	0.67	0.502	F	0.96	0.916	0
Trp vs. Arg	Overall	21	1.69(1.46–1.95)	7.03	<0.001	R	33.41	0.03	40.1
	QS								
	High quality	16	1.67(1.39–1.99)	5.61	<0.001	R	31.47	0.008	52.3
	Low quality	5	1.78(1.36–2.33)	4.18	<0.001	R	1.66	0.798	0
Overall survival(HR)									
TrpTrp vs. ArgArg	Overall	5	0.84(0.64–1.11)	1.22	0.223	F	5.59	0.232	28.4
TrpArg vs. ArgArg	Overall	5	0.96(0.79–1.16)	0.45	0.653	F	2.05	0.727	0
TrpTrp+TrpArg vs. ArgArg	Overall	3	0.93(0.72–1.21)	0.54	0.590	F	0.46	0.795	0
Median progression-free survival(HR)									
TrpTrp vs. ArgArg	Overall	3	1.01(0.69–1.48)	0.07	0.948	F	2.17	0.338	7.8
TrpArg vs. ArgArg	Overall	3	1.06(0.82–1.36)	0.44	0.662	F	0.49	0.782	0
TrpTrp+TrpArg vs. ArgArg	Overall	2	1.05(0.79–1.38)	0.31	0.753	F	0.89	0.346	0

Note: OR, odds ratio; HR: hazard ratio; CI, confidence interval; vs., versus; QS: quality score; TrpTrp vs. ArgArg: Homozygote comparison; TrpArg vs. ArgArg: Heterozygote comparison; TrpTrp+TrpArg vs. ArgArg: Dominant model; TrpTrp vs. TrpArg+ArgArg: Recessive model; Trp vs. Arg: Allele contrast; F, fixed effect model; R, random effect model; Random effect model was chosen when P-value < 0.10 and/or I^2^ > 50% for heterogeneity test; otherwise fixed effect model was used.

^a^The detailed references are given in Table 1.

^b^The OR for objective response rate.

^c^The HR for overall survival and median progression-free survival.

**Table 4:** Meta-analysis of the association between *XRCC1* Arg399Gln polymorphism and platinum-based chemotherapy in objective response rate, overall survival and median progression-free survival for advanced lung cancer patients.

Genetic comparisons	Study groups	No. ofstudies^a^	Test of association		P-value	Model	Test of heterogeneity		
			OR^b^/HR^c^ (95% CI)	Z			χ^2^	P-value	I^2^(%)
Objective response rate(OR)									
GlnGln vs. ArgArg	Overall	26	0.75(0.47–1.20)	1.22	0.223	R	77.93	<0.001	67.9
	Populations								
	Asian	23	0.68(0.39–1.18)	1.38	0.169	R	74.2	<0.001	70.4
	Caucasian	3	1.06(0.50–2.24)	0.14	0.889	R	3.61	0.165	44.6
	QS								
	High quality	20	0.85(0.51–1.42)	0.61	0.541	R	69.57	<0.001	72.7
	Low quality	6	0.36(0.14–0.94)	2.08	0.037	R	1.55	0.907	0
GlnArg vs. ArgArg	Overall	26	0.80(0.62–1.02)	1.79	0.074	R	66.61	<0.001	62.5
	Populations								
	Asian	23	0.80(0.60–1.06)	1.57	0.117	R	64.46	<0.001	65.9
	Caucasian	3	0.73(0.48–1.09)	1.54	0.123	R	0.34	0.844	0
	QS								
	High quality	20	0.86(0.66–1.13)	1.08	0.278	R	52.26	<0.001	63.6
	Low quality	6	0.50(0.24–1.02)	1.91	0.057	R	9.89	0.078	49.5
GlnGln+GlnArg vs. ArgArg	Overall	38	0.68(0.54–0.86)	3.20	0.001	R	130.81	<0.001	71.7
	Populations								
	Asian	33	0.65(0.50– 0.86)	3.03	0.002	R	130.43	<0.001	75.50
	Caucasian	5	0.80(0.58–1.10)	1.41	0.158	R	0.17	0.996	0
	QS								
	High quality	28	0.72(0.56–0.94)	2.4	0.017	R	106.56	<0.001	74.7
	Low quality	10	0.53(0.32–0.89)	2.39	0.017	R	19.08	0.025	52.8
GlnGln vs. GlnArg+ArgArg	Overall	26	0.85(0.58–1.25)	0.81	0.415	R	59.09	<0.001	57.7
	Populations								
	Asian	23	0.78(0.50–1.21)	1.11	0.268	R	54.3	<0.001	59.5
	Caucasian	3	1.21(0.54–2.70)	0.46	0.647	R	4.75	0.093	57.9
	QS								
	High quality	20	0.92(0.60–1.40)	0.41	0.685	R	54.83	<0.001	65.3
	Low quality	6	0.48(0.19–1.21)	1.56	0.119	R	0.27	0.998	0
Gln vs. Arg	Overall	26	0.80(0.63–1.01)	1.87	0.061	R	119.97	<0.001	79.2
	Populations								
	Asian	23	0.78(0.59–1.01)	1.87	0.062	R	117.97	<0.001	81.4
	Caucasian	3	0.94(0.72–1.24)	0.43	0.670	R	1.65	0.438	0
	QS								
	High quality	20	0.86(0.66–1.11)	1.15	0.251	R	105.25	<0.001	81.9
	Low quality	6	0.62(0.43–0.90)	2.51	0.012	R	6.11	0.296	18.2
Overall survival(HR)									
GlnGln vs. ArgArg	Overall	14	1.14(0.75–1.75)	0.62	0.533	R	58.1	<0.001	77.6
	Populations								
	Asian	10	0.90(0.57–1.43)	0.43	0.666	R	29.53	0.001	69.5
	Caucasian	4	1.91(0.77–4.73)	1.4	0.161	R	21.45	<0.001	86
GlnGln+GlnArg vs. ArgArg	Overall	8	0.84(0.64–1.09)	1.33	0.183	R	20.56	0.004	65.9
	Populations								
	Asian	7	0.81(0.60–1.09)	1.38	0.166	R	20.19	0.003	70.3
	Caucasian	1	1.01(0.66–1.55)	0.05	0.963	R	—	—	—
	QS								
	High quality	7	0.85(0.63–1.15)	1.08	0.282	R	19.58	0.003	69.3
	Low quality	1	0.74(0.50–1.10)	1.48	0.139	R	20.56	0.004	65.9
GlnArg vs. ArgArg	Overall	13	0.91(0.75–1.10)	0.96	0.337	R	32.21	0.001	62.7
	Populations								
	Asian	9	0.84(0.66–1.08)	1.38	0.166	R	23.55	0.003	66
	Caucasian	4	1.07(0.77–1.47)	0.38	0.701	R	6.93	0.074	56.7
GlnGln vs. GlnArg	Overall	3	1.42(1.01–2.00)	2.03	0.043	F	0.67	0.714	0
	Populations								
	Asian	1	1.20(0.71–2.03)	0.68	0.498	F	—	—	—
	Caucasian	2	1.60(1.03–2.50)	2.08	0.038	F	0	0.978	0
Median progression-free survival(HR)									
GlnGln vs. ArgArg	Overall	4	0.80(0.58–1.11)	1.34	0.179	F	3.53	0.317	14.9
	Populations								
	Asian	3	0.72(0.48–1.08)	1.58	0.114	F	2.83	0.243	29.2
	Caucasian	1	0.97(0.55–1.70)	0.11	0.915	F	—	—	—
GlnArg vs. ArgArg	Overall	3	0.91(0.71–1.17)	0.73	0.468	F	1.96	0.376	0
GlnGln+GlnArg vs. ArgArg	Overall	4	0.86(0.70–1.06)	1.38	0.166	F	1.69	0.638	0
	Populations								
	Asian	3	0.87(0.68–1.12)	1.09	0.277	F	1.65	0.438	0
	Caucasian	1	0.83(0.55–1.26)	0.88	0.378	F	—	—	—

Note: OR, odds ratio; HR: hazard ratio; CI, confidence interval; vs., versus; QS: quality score; GlnGln vs. ArgArg: Homozygote comparison; GlnArg vs. ArgArg: Heterozygote comparison; GlnGln+GlnArg vs. ArgArg: Dominant model; GlnGln vs. GlnArg+ArgArg: Recessive model; Gln vs. Arg: Allele contrast; F, fixed effect model; R, random effect model; Random effect model was chosen when P-value < 0.10 and/or I^2^ > 50% for heterogeneity test; otherwise fixed effect model was used.

^a^The detailed references are given in Table 1.

^b^The OR for objective response rate.

^c^The HR for overall survival and median progression-free survival.

**Table 5:** The scale for quality assessment.

Criteria	Item	Score
Evaluation criteria		
	WHO/RECIST	3
	Not described	0
Platinum combinations		
	One kind of platinum combinations	3
	TAX/TXT, DOC, GEM, or NVB	2
	Not detailed or other regimens	1
Clinical stage		
	Detailed	3
	Not detailed	0
Overall survival		
	Original data	3
	Estimation from the Kaplan–Meier curves	1
	Not described	0
Median survival time		
	Original data	3
	Not described	0
Median PFS		
	Original data	3
	Estimation from the Kaplan–Meier curves	1
	Not described	0
Genotyping methods		
	3D DNA microarray	3
	DNA microarray	3
	DNA Sequencing	3
	Illumina Golden Gate Platform	3
	MALDI-TOF-MS	3
	MassARRAY	3
	Sequenome MS-based genotyping assay	3
	SNPstream UHT	3
	TaqMan	3
	PCR-LDR	2
	PCR-CTPP	2
	PCR-CTTP	2
	PCR-RFLP	2
Total sample size		
	≥150	3
	>100 but <150	2
	≤100	1

Note: WHO, World Health Organization; RECIST, Response Evaluation Criteria in Solid Tumors; TAX, paclitaxel; TXT, docetaxel; DOC, docetaxel ; GEM, gemcitabine; NVB, vinorelbine; PFS, progression-free survival; 3D, 3-dimensional; MALDI-TOF-MS, matrix-assisted laser desorption/ionization time of light mass spectrometry; Sequenome MS-based genotyping assay, sequenome mass spectrometry-based genotyping assay; SNPstream UHT, SNPstream ultra high throughput; PCR-LDR, polymerase chain reaction (PCR)-ligase detection reaction; PCR-CTPP, duplex PCR with the confronting-two-pair primer; PCR-CTTP, PCR with confronting two-pair primers; PCR-RFLP, PCR-restriction fragment length polymorphism.
